# IL-6 trans-signaling in a humanized mouse model of scleroderma

**DOI:** 10.1073/pnas.2306965120

**Published:** 2023-09-05

**Authors:** Ian D. Odell, Kriti Agrawal, Esen Sefik, Anahi V. Odell, Elizabeth Caves, Nancy C. Kirkiles-Smith, Valerie Horsley, Monique Hinchcliff, Jordan S. Pober, Yuval Kluger, Richard A. Flavell

**Affiliations:** ^a^Department of Dermatology, Yale University School of Medicine, New Haven, CT 06520; ^b^Department of Immunobiology, Yale University School of Medicine, New Haven, CT 06520; ^c^Program in Computational Biology and Bioinformatics, Yale University, New Haven, CT 06511; ^d^Program in Applied Mathematics, Yale University, New Haven, CT 06511; ^e^Department of Molecular, Cellular, and Developmental Biology, Yale University, New Haven, CT 06520; ^f^Department of Internal Medicine, Section of Rheumatology, Allergy and Immunology, Yale School of Medicine, New Haven, CT 06520; ^g^Department of Pathology, Yale University, New Haven, CT 06511; ^h^HHMI, Chevy Chase, MD 20815

**Keywords:** scleroderma, systemic sclerosis, fibrosis, humanized mice, interleukin 6

## Abstract

Fibrosis is regulated by interactions between immune and mesenchymal cells. However, the capacity of cell types to modulate human fibrosis pathology is poorly understood due to lack of a fully humanized model system. MISTRG6 mice were engineered by homologous mouse/human gene replacement to develop an immune system like humans when engrafted with human hematopoietic stem cells (HSCs). We utilized MISTRG6 mice to model scleroderma by transplantation of healthy or scleroderma skin from a patient with pansclerotic morphea to humanized mice engrafted with unmatched allogeneic HSC. We identified that scleroderma skin grafts contained both skin and bone marrow–derived human CD4 and CD8 T cells along with human endothelial cells and pericytes. Unlike healthy skin, fibroblasts in scleroderma skin were depleted and replaced by mouse fibroblasts. Furthermore, HSC engraftment alleviated multiple signatures of fibrosis, including expression of collagen and interferon genes, and proliferation and activation of human T cells. Fibrosis improvement correlated with reduced markers of T cell activation and expression of human IL-6 by mesenchymal cells. Mechanistic studies supported a model whereby IL-6 trans-signaling driven by CD4 T cell–derived soluble IL-6 receptor complexed with fibroblast-derived IL-6 promoted excess extracellular matrix gene expression. Thus, MISTRG6 mice transplanted with scleroderma skin demonstrated multiple fibrotic responses centered around human IL-6 signaling, which was improved by the presence of healthy bone marrow–derived immune cells. Our results highlight the importance of IL-6 trans-signaling in pathogenesis of scleroderma and the ability of healthy bone marrow–derived immune cells to mitigate disease.

## Significance

Scleroderma is an autoimmune disease that causes skin and internal organ fibrosis. No mouse model has been identified that fully recapitulates human disease. Therefore, we tested whether the MISTRG6 strain of humanized mice could be transplanted with healthy or scleroderma human skin grafts. We found that healthy and scleroderma skin grafts retained skin and bone marrow–derived immune cells. Moreover, fibrosis in scleroderma skin grafts was alleviated by engraftment with unmatched allogeneic hematopoietic stem cells. Mechanistic studies supported a trans-signaling model whereby CD4 T cell–derived soluble interleukin-6 (IL-6) receptor complexed with interferon-driven IL-6 cytokine drives skin fibrosis. Thus, scleroderma skin transplants to MISTRG6 humanized mice recapitulate a key signaling pathway of scleroderma and may be a useful model to study human disease.

Scleroderma is an umbrella term that incorporates patients with systemic sclerosis (SSc), an immune-mediated disease that causes fibrosis and vasculopathy of the skin and internal organs (1, 2), and morphea (also called localized scleroderma), that causes skin fibrosis without internal organ involvement. Skin gene expression studies of scleroderma patients showed similar expression profiles between morphea and an inflammatory subset of SSc, suggesting shared pathogenic processes (3). The most severe form of localized scleroderma is pansclerotic morphea, which causes fibrosis of all layers of the skin, and the childhood form was recently found to have elevated IL-6 expression regulated by STAT4 gain of function mutations (4). As with most human autoimmune diseases, scleroderma has complex genetic contribution (5–8), and its pathogenesis involves several immune and mesenchymal cell types. Immune cells such as monocytes (9, 10), plasmacytoid dendritic cells (11, 12), and type 2 innate lymphoid cells (13) communicate by incompletely understood mechanisms with fibroblasts and pericytes to regulate expression of collagen and other extracellular matrix (ECM) genes. A better mechanistic understanding of scleroderma requires in vivo disease models that can emulate the cellular composition and regulatory signals that occur in human patients.

Investigation of scleroderma in mice is hindered by a lack of mouse models that fully recapitulate the complexity of human disease. To model the human immune system in mice, human-specific growth factors and cytokines are required for proper immune cell development from the bone marrow. We generated the MISTRG6 humanized mouse strain (14) to provide physiologic expression of human *CSF1* (monocytes and tissue macrophage development) (15), *CSF2/IL3* (lung alveolar macrophages) (16), *SIRPA* (macrophage tolerance to human cells) (17), *THPO* (hematopoiesis and platelets) (18), and *IL6* (improved engraftment and antibody responses) (19) knocked into their respective mouse loci on a *Rag2^-/-^Il2rg^-/-^* background, thereby permitting development of both innate and adaptive lineages from engrafted human hematopoietic stem cells (HSCs). As both adaptive and innate immune cells are important regulators of fibrosis, the development of a comprehensive human immune repertoire by MISTRG6 mice may provide a highly suited system to model those interactions in scleroderma.

To fully model immune–mesenchymal interactions in mice, HSC-engrafted mice require the presence of human tissue, such as by transplantation of human skin. However, skin grafts alone do not contain a full panoply of bone marrow–derived human immune cells, which may be important regulators of disease. We hypothesized that engrafting both human HSC and skin to MISTRG6 mice would allow examination of key immune–mesenchymal interactions in scleroderma. We identified that MISTRG6 mice could be stably engrafted with human skin and contained a broad range of human immune and mesenchymal cell types. Skin grafts from a patient with pansclerotic morphea to HSC-engrafted MISTRG6 mice demonstrated that healthy bone marrow–derived T cells were able to improve pathologic signatures of fibrosis including ECM gene expression and T cell activation. These findings underscore the importance of bone marrow–derived immune cells in regulating fibrotic skin disease.

## Results

### Healthy Human Skin Grafting onto MISTRG Mice.

**Fig. 1.**
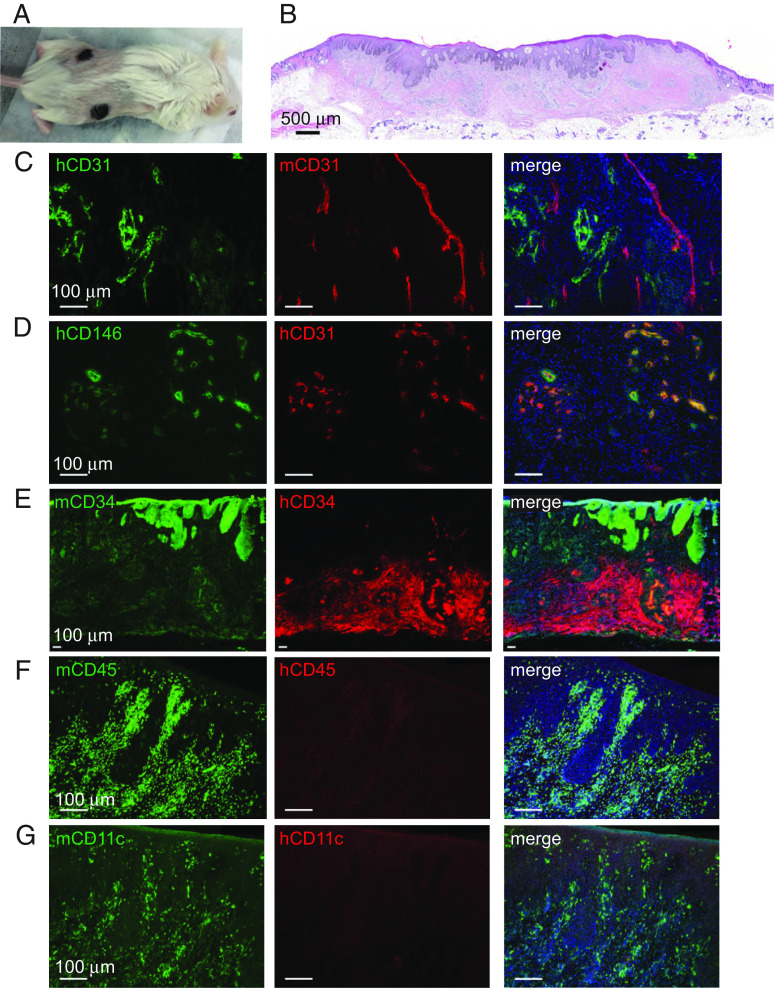
Human skin grafting onto MISTRG mice. (*A*) MISTRG mice were transplanted with STSG to the dorsal flanks, visible by the brown pigmentation. (*B*) Histology of a representative skin graft after transplantation for 7 wk. (*C*–*G*) Immunofluorescence images of skin grafts with species-specific antibodies against endothelial cells (*C*), pericytes (*D*), fibroblasts (*E*), and immune cells (*F* and *G*). Slides were imaged with a Keyence BZ-X800 microscope (*B* and *E*) or Leica DMI6000 B Microscope (*C*, *D*, *F*, and *G*). Images are all 10× magnification, and lower power images in *B* and *E* were digitally stitched together.

Acceptance of human HSC and vascularized skin grafts varies by immunodeficient mouse strains, in particular, whether innate immune responses reject certain cell types and/or tissues (20). To determine what human cell types are retained in skin grafts to MISTRG mice, we transplanted split-thickness skin grafts (STSG) of healthy human skin onto the backs of 8-wk-old MISTRG mice. Staples were removed after 14 d, and the grafts were allowed to heal and mature for a total of 7 wk. As shown in Fig. 1*A*, two skin grafts were transplanted to each mouse and were readily apparent by their brown pigmentation because the skin donor was of African American ancestry. The skin grafts appeared viable and intact on histology (Fig. 1*B*). Typical findings of STSG were observed including epidermal hyperplasia, absence of hair follicles, and degeneration of sebaceous and eccrine glands (21). Also typical of STSG, modest contracture (22) of the graft area occurred. Overall, skin grafts from healthy human skin to MISTRG mice showed good viability and stability in the first 7 wk.

To ascertain what human and mouse cell types were present in the skin grafts, we obtained immunofluorescence images using human and mouse-specific antibodies. Both human and mouse vasculature were present in skin graft dermis by the presence of CD31^+^ endothelial cells (Fig. 1*C*). Mouse vessels coursed through the entire thickness of the dermis intermixed between the human blood vessels. Human vessels retained their associated pericytes detected by CD146^+^ staining (Fig. 1*D*). The graft dermis also showed the presence of human fibroblasts by CD34^+^ positivity, which was sharply demarcated from underlying murine fascia (Fig. 1*E*). Mouse myeloid immune cells infiltrated both the dermal and epidermal compartments of the skin graft (Fig. 1*F*), which included CD11c^+^ dendritic cells and macrophages (Fig. 1*G*). However, we did not observe human immune cells within skin grafts to MISTRG mice by immunofluorescence imaging. In summary, healthy human skin grafts to MISTRG mice are viable and retain multiple human mesenchymal cell populations including fibroblasts, endothelial cells, and pericytes alongside multiple murine structural and immune cell types.

### Healthy Human Skin Retains T Cells Long Term in MISTRG6 Mice.

**Fig. 2.**
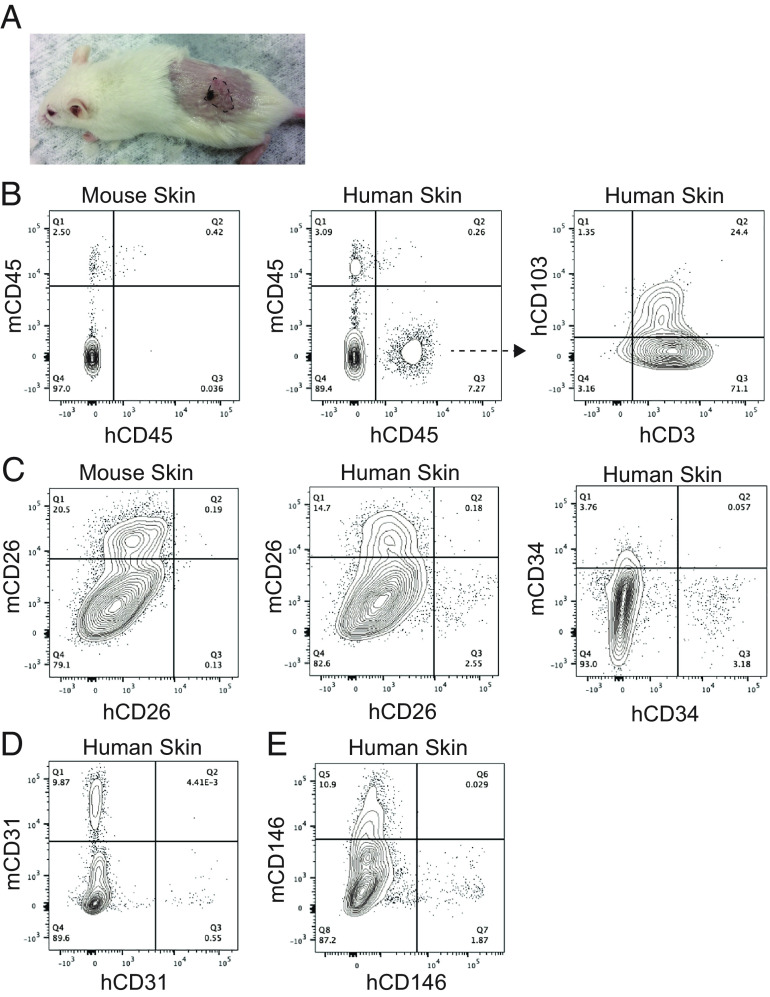
Healthy human skin retains T cells long term in MISTRG6 mice. (*A*) MISTRG6 mice were transplanted with STSG to the dorsal flanks and followed for 9 mo. Residual graft is visible by brown pigmentation and highlighted with the dotted line. (*B*–*E*) Skin grafts transplanted for 9 mo were digested to create single-cell suspension and analyzed by FACS for human and mouse immune and mesenchymal cell populations.

The presence of the human IL-6 gene in the MISTRG strain of humanized mice (MISTRG6) dramatically improves HSC engraftment and enhances B cell development (19). We considered whether human IL-6 would modify skin graft cellular constituents and graft longevity. We transplanted STSG of healthy human skin from a second healthy donor this time to MISTRG6 mouse recipients, and mice were allowed to age up to an additional 9 mo after transplantation. Even after 9 mo, skin grafts were readily visible by the naked eye and contained some of the original skin pigmentation (Fig. 2*A*). To assess immune and mesenchymal cells present in the skin grafts, we digested the grafts to generate single-cell suspensions and analyzed the cellular components by fluorescence-activated cell sorting (FACS) compared to MISTRG6 mouse control skin. In contrast to MISTRG mice, skin grafts on MISTRG6 mice retained human immune cells in the skin, which consisted almost entirely of T cells (Fig. 2*B*). Given their long-term retention in human skin and absence in control MISTRG6 mouse skin, the T cells were likely tissue resident. Accordingly, at least ¼ of the T cells showed positive CD103 staining on FACS, as CD103 mediates adhesion to the skin epidermis. In addition to human T cells, skin grafts retained human fibroblasts, detected by CD26 and CD34 staining (Fig. 2*C*), human endothelial cells marked by CD31 (Fig. 2*D*), and human pericytes positive for CD146 (Fig. 2*E*). Therefore, MISTRG6 mice support healthy human skin grafts with long-term retention of human tissue-resident T cells as well as fibroblasts, endothelial cells, and pericytes for at least up to 9 mo after engraftment.

### Transplantation of Scleroderma Skin to MISTRG6 Mice.

**Fig. 3.**
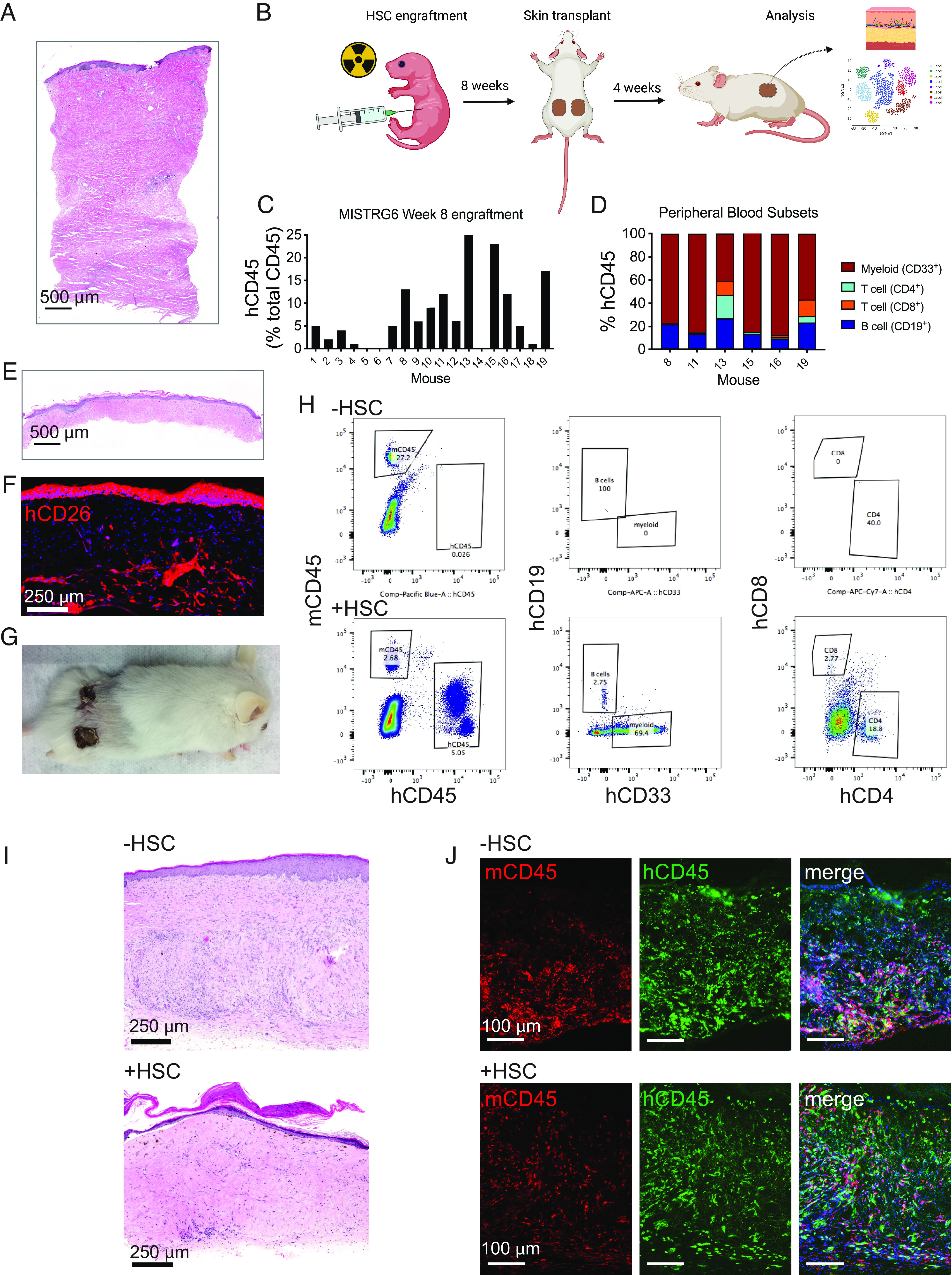
Transplantation of scleroderma skin to MISTRG6 mice. (*A*) Histology of punch biopsy from scleroderma skin donor. (*B*) Diagram of the experimental setup. (*C*) Human peripheral blood immune cell engraftment levels of 8-wk-old prospective MISTRG6 skin transplant recipients. (*D*) Peripheral blood immune cell proportions of highest engrafted MISTRG6 mice. (*E*) Histology of STSG from the scleroderma donor at the same magnification as *A*. (*F*) Immunofluorescence imaging of STSG with the fibroblast marker CD26. (*G*) Scleroderma skin grafts on euthanized MISTRG6 mouse at completion of the experiment. (*H*) Peripheral blood human immune cells of MISTRG6 mice with scleroderma skin comparing HSC engrafted (+HSC) and unengrafted (−HSC) mice. (*I*) Histology of scleroderma skin grafts to HSC-unengrafted and -engrafted MISTRG6 mice. (*J*) Scleroderma skin graft immune cell staining by CD45 comparing HSC-engrafted and -unengrafted mice. Histology and immunofluorescent images were obtained with a Keyence BZ-X800 microscope. Low-power images are 10× magnification and stitched together.

Scleroderma skin has multiple characteristics that could make skin transplantation difficult, such as increased stiffness and dysregulated metabolism (23). We report a patient with adult-onset pansclerotic morphea, a severe subtype of localized scleroderma that affects all layers of the skin but spares internal organs, who was highly motivated to enroll in our study and donate STSG for transplantation to humanized mice. Our patient had long-standing progressive disease despite therapy with prednisone, cyclosporine, cyclophosphamide, thalidomide, tocilizumab, and extracorporeal photopheresis (ECP). Ongoing therapy at the time of skin grafting was monthly sessions of ECP, most recently done 1 wk prior to skin graft. A baseline punch biopsy of his skin is shown in Fig. 3*A*, demonstrating full-thickness fibrosis of the entire dermal layer. We prepared a cohort of MISTRG6 recipient mice by injecting 19 neonatal MISTRG6 mice intrahepatically each with 10,000 CD34^+^ HSC (Fig. 3*B*). Peripheral blood was monitored at 6 and 8 wk postengraftment for immune reconstitution. The six MISTRG6 mice with the most favorable immune engraftment (#8, 11, 13, 15, 16, and 19) were selected as recipients for patient skin grafts (Fig. 3*C*) and contained a high percentage of the myeloid lineage (Fig. 3*D*). At 8 wk after HSC engraftment, a 0.75-mm-thick STSG (Fig. 3*E*) was obtained from the patient’s back, cut into ~1 cm^2^ squares, and transplanted two per mouse onto the backs of 6 HSC-engrafted MISTRG6 mice and 4 unengrafted control MISTRG6 mice. Because the skin dermis contains spatially distributed populations of fibroblasts (24), we confirmed that profibrotic fibroblasts marked by CD26 were present in the donor skin grafts (Fig. 3*F*) (25). The skin grafts were allowed to heal and mature for 4 wk (Fig. 3*G*), at which point the mice were euthanized and skin grafts analyzed for histology and scRNA-Seq.

The effects of HSC engraftment in combination with skin transplantation could each contribute to the presence of circulating and tissue immune cells. To interrogate circulating immune cells, we performed FACS analysis of peripheral blood of HSC-engrafted (+HSC) MISTRG6 mice compared to unengrafted (−HSC) control MISTRG6 mice. We observed circulating myeloid and adaptive immune subsets only in the HSC-engrafted mice which were absent in all the unengrafted controls (Fig. 3*H*). This signifies that human skin graft–derived immune cells did not detectably populate the blood. To investigate the presence of immune cells in the skin tissue, we assessed skin histology and immunofluorescence images of skin grafts. By histology, scleroderma skin grafts on HSC-unengrafted MISTRG6 mice showed a prominent cellular infiltrate in the dermis similar to what we observed with healthy skin on MISTRG mice (Fig. 3 *I*, *Upper*). In contrast, scleroderma skin grafts of HSC-engrafted mice showed less cellularity, suggesting fewer numbers of one or more cell types (Fig. 3 *I*, *Lower*). Nonetheless, human scleroderma skin of both HSC-engrafted and -unengrafted mice contained numerous human and mouse immune cells labeled by CD45 (Fig. 3*J*). This observation suggests that like healthy skin grafts, scleroderma skin grafts retained resident human immune cells in MISTRG6 mice. Therefore, scleroderma skin grafts contained a chimeric assortment of mouse and human immune cells of skin and bone marrow origin. In contrast, skin-derived human immune cells did not circulate in the blood to an appreciable level.

### scRNA-Seq Analysis of Transplanted Scleroderma Skin.

**Fig. 4.**
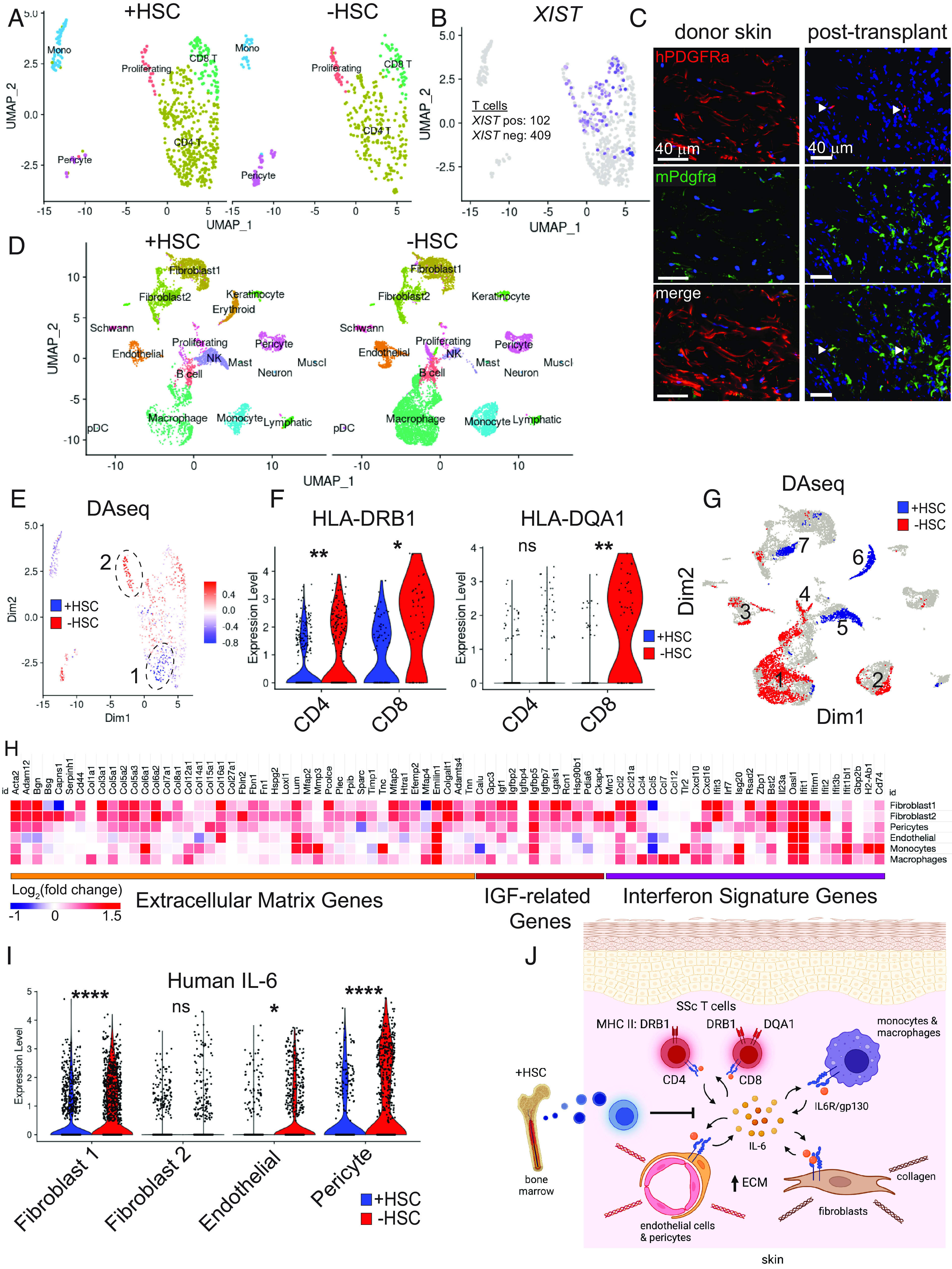
scRNA-Seq analysis of transplanted scleroderma skin. (*A*) UMAP embedding of human cell populations in scleroderma skin grafts comparing HSC engrafted (+HSC) to unengrafted mice. (*B*) Human XIST expression of HSC-engrafted mice to distinguish bone marrow–derived immune cells. (*C*) Immunofluorescence images of scleroderma skin graft before and after transplantation of human and mouse PDGFRA. Arrowheads indicate human fibroblasts in right panels. (*D*) UMAP embedding of mouse cell populations in scleroderma skin grafts. (*E*) DAseq regions of human cells. (*F*) Expression of MHC class II genes by human CD8 T cells. (*G*) DAseq regions of mouse cells. (*H*) Heatmap of differentially expressed genes with *P* value <0.05 by mouse fibroblasts, pericytes, endothelial cells, monocytes, and macrophages. Fold change refers to ratio unengrafted/HSC engrafted. (*I*) Violin plot of human IL-6 expression by mouse mesenchymal cell types. (*J*) Model of human T cell interactions with mouse myeloid and mesenchymal cells. IL-6 expression by multiple cell types induces expression of MHC class II molecules on human T cells along with elevated ECM expression, which is down-regulated by healthy bone marrow–derived immune cells. Data in *F*, *H*, and *I* are analyzed with a nonparametric Wilcoxon rank-sum test adjusted with Bonferroni correction using all features in the dataset, with *P < 0.05, **P < 0.01, ***P < 0.001, and ****P < 0.0001. Immunofluorescent images were obtained with a Keyence BZ-X800 microscope at 40× magnification.

To understand how skin and bone marrow–derived human immune cells alter the cellular signaling in scleroderma skin grafts, we performed scRNA-Seq on 3 of the HSC-engrafted (+HSC) compared to 3 HSC-unengrafted (−HSC) scleroderma skin grafts 4 wk after transplantation onto MISTRG6 humanized mice. Immediately after euthanasia of the mice, the skin grafts were excised and digested to create single-cell suspensions. Live cells were sorted and processed with the 10× Chromium Single Cell Controller to create barcoded single-cell cDNA libraries, and sequencing reads were aligned to mouse and human genomes. Because MISTRG6 mice express human transgenes by mouse cells, we also needed to align reads to a combined human and mouse genome to assess expression of human genes by mouse cells. Uniform Manifold Approximation and Projection (UMAP) embedding of the single-cell cDNA identified 5 cell clusters of human cells, including CD4 and CD8 T cells, monocytes, pericytes, and a population of proliferating cells (Fig. 4*A*). Bone marrow–derived T cells could be identified by expression of the long noncoding RNA *XIST* due to the sex mismatch of HSC and skin donors (female HSC, male scleroderma skin) (Fig. 4*B*). This showed that 20% of the human CD4 and CD8 T cells in the HSC-engrafted mice were bone marrow derived, whereas the other 80% were retained from the original human skin. Unexpectedly, no human fibroblasts were detected in our scRNA-Seq data of scleroderma skin grafts independent of HSC engraftment. The absence of human fibroblasts in scleroderma skin grafts was corroborated by immunofluorescence imaging of scleroderma skin grafts before and after transplantation using human and mouse PDGFRA antibodies (Fig. 4*C*). Prior to skin transplantation, human fibroblasts were apparent in the scleroderma donor skin, whereas 4 wk posttransplantation, scleroderma skin grafts were largely devoid of human fibroblasts with only rare human PDGFRA positive cells (arrowheads) and were replaced by mouse fibroblasts. Thus, scleroderma skin grafts retained human CD4 and CD8 T cells and pericytes but lost human fibroblasts.

In the scRNA-Seq data that aligned to the mouse genome, we observed 15 clusters of mouse cells (Fig. 4*D*). Murine cell types included two clusters of fibroblasts as well as pericytes, endothelial cells (both vascular and lymphatic), monocytes, and macrophages, and smaller groups of neurons, Schwann cells, muscle, and mast cells. In the scleroderma skin grafts, mouse cells predominated in number with 13,805 mouse cells compared to 1,057 human cells. Engraftment with human HSC associated with different proportions of mouse cell populations in scleroderma skin grafts. Scleroderma skin grafts of HSC-engrafted mice showed 70% fewer mouse monocytes and macrophages and a new population of mouse erythroid cells. Although the erythroid cells were merely due to anemia secondary to HSC engraftment (14), the alterations in myeloid populations may reflect differences in immune–fibroblast signaling. Altogether, our findings demonstrated that human scleroderma skin grafts contained human resident and bone marrow–derived T cells and were also infiltrated with mouse mesenchymal and myeloid immune cells.

To identify gene expression modules altered in scleroderma skin grafts by bone marrow–derived immune cells in an unbiased manner, we analyzed our scRNA-Seq data for differentially abundant (DA) cell subpopulations using DA-Seq (26). This approach detects cell subpopulations whose abundance differs between two states. We observed two DA regions in the human cells and seven DA regions in the mouse cells. Among human T cells, DA region 1 highlighted an increased abundance of skin CD4 T cells in HSC-engrafted mice in blue, which contrasts with relatively more abundant CD8 T cells in HSC-unengrafted mice in red (Fig. 4*E*). Human DA region 2 highlighted proliferating T cells, which may reflect local expansion of T cells in HSC-unengrafted mice. To understand what genes might be driving these observations, we calculated differential gene expression of HSC-engrafted vs. -unengrafted human CD4 and CD8 T cells. We identified decreased expression of MHC class II genes by both CD4 and CD8 T cells in HSC-engrafted compared to -unengrafted mice (Fig. 4*F*). The differential expression of these genes was not affected by the presence of bone marrow–derived (XIST positive) T cells. Notably, the differentially expressed MHC class II alleles we observed are significant predictors of mortality in scleroderma patients (27). Although the function of MHC class II expression by human T cells is not entirely clear, it is a well-established marker of T cell activation (28). Thus, loss of MHC II expression by scleroderma T cells suggests decreased T cell activation in HSC-engrafted samples. Altogether, our results suggest that activation of scleroderma skin–derived CD4 and CD8 T cells is decreased by HSC engraftment.

Among mouse cells, DA regions of HSC-engrafted mice highlighted a decrease in monocytes, macrophages, and endothelial cells (regions 1 to 3) and a region of increased fibroblasts (region 7) (Fig. 4*G*). Proliferating cells were again highlighted in HSC-unengrafted mice (region 4), as well as erythroid cells in HSC-engrafted mice (region 6). NK cells in region 5 were highlighted, but these were disproportionally present in only one of the three scleroderma skin grafts, so were excluded from further analysis. To understand the gene expression changes associated with the DA regions, we calculated differential gene expression of cells from skin grafts of HSC-engrafted vs. -unengrafted mice followed by pathway analysis. In HSC-unengrafted mice, we observed significantly up-regulated expression of the myofibroblast marker aSMA (Acta2) by fibroblasts along with increased expression of many ECM genes by fibroblasts, pericytes, and endothelial cells (Fig. 4*H*). These same cell types showed upregulation of insulin growth factor related genes, another profibrotic signaling pathway (29). Multiple cell types in skin grafts of HSC-unengrafted mice, including fibroblasts, pericytes, endothelial cells, monocytes, and macrophages, also showed elevated expression of interferon signature genes, which are associated with scleroderma severity (30, 31). Therefore, scleroderma skin grafts of HSC-unengrafted mice showed a fibrotic expression signature by murine cells including higher ECM expression, growth factor signaling, and interferon response.

We considered what signal might be driving the array of cellular alterations in scleroderma skin, including activation and proliferation of human T cells along with elevated ECM and ISG expression. The profibrotic cytokine IL-6 has been linked to each of these observations including induction of T cell proliferation and activation (32), induction of collagen expression by dermal fibroblasts (33), and regulation by type I interferon (34). IL-6 is elevated in the skin and blood of patients with localized scleroderma and SSc (35, 36) and was recently identified to be overexpressed by fibroblasts of patients with childhood-onset pansclerotic morphea (4). We therefore assessed the effects of HSC engraftment on human IL-6 expression in the scleroderma xenografts. We found that HSC-unengrafted mice had significantly higher expression of human IL-6 by mouse fibroblasts, endothelial cells, and pericytes (Fig. 4*I*), which corresponds with their activated and proliferating T cells and elevated ECM expression compared to HSC-engrafted mice. Elevated expression of human IL-6 thus links the T cell and mesenchymal changes characteristic of fibrotic scleroderma skin. Altogether, the up-regulated transcriptional programs in scleroderma skin of HSC-unengrafted compared to HSC-engrafted mice demonstrated a more fibrotic program through elevated expression of multiple fibrosis signature genes, including aSMA, ECM, and interferon response, which all center around elevated expression of human IL-6 (Fig. 4*J*).

### IL-6 Trans-Signaling between CD4 T Cells and Fibroblasts.

**Fig. 5.**
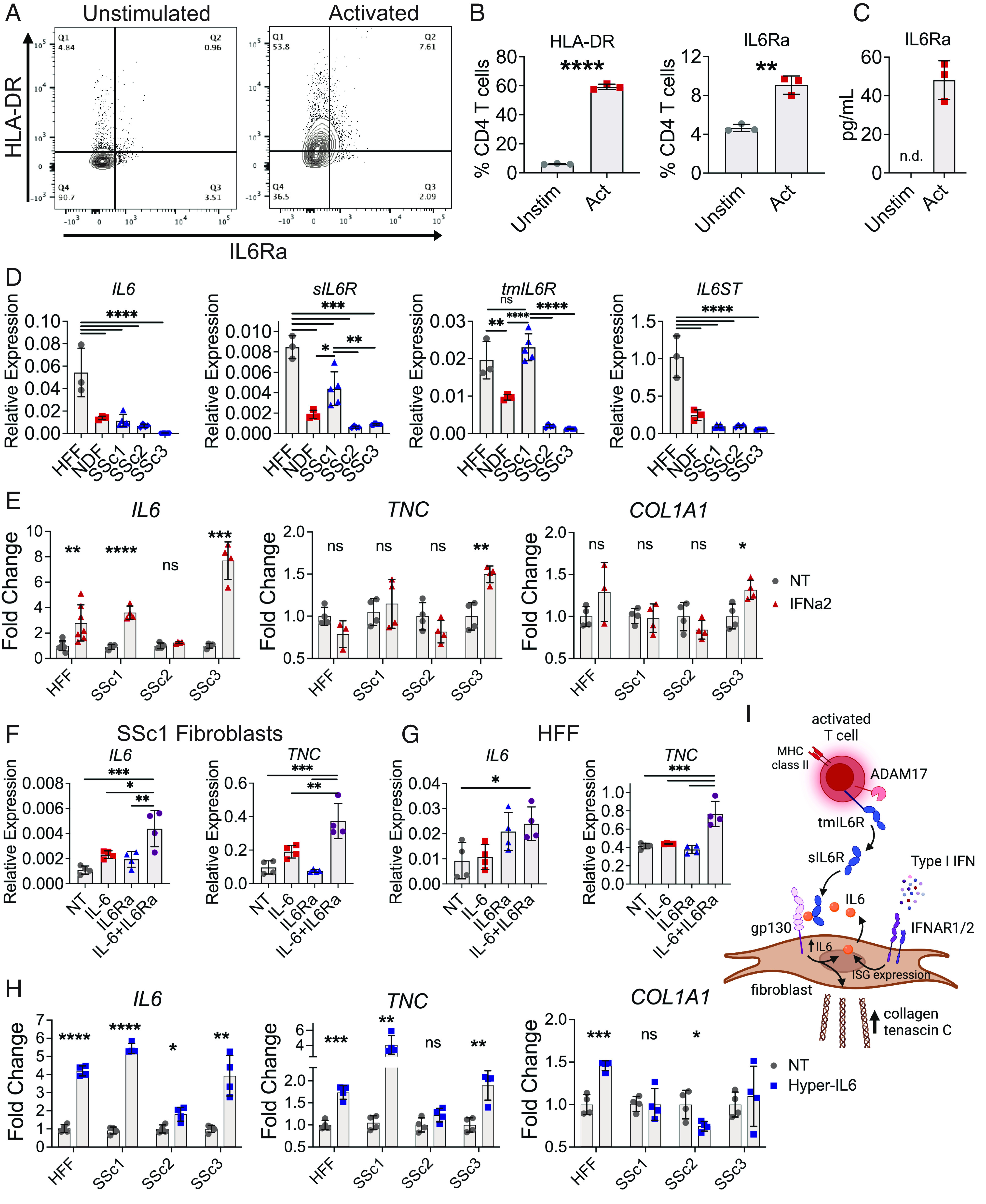
IL-6 trans-signaling drives ECM expression in fibroblasts. (*A*–*C*) CD4^+^ T cells from peripheral blood were activated with CD3/CD28 Dynabeads for 3 d, n = 3 per group; data are representative of 2 independent experiments. (*A*) Representative FACS plot of HLA-DR and IL6Ra staining. (*B*) Quantification of HLA-DR and IL6Ra expression by CD4^+^ T cells. (*C*) sIL6Ra levels in CD4 T cell supernatant, n.d. not detected. (*D*) Relative expression of IL-6 signaling genes in fibroblasts, n = 3 to 5 per group. (*E*) *IL6* expression by HFF and SSc fibroblasts incubated with IFNa2, n = 7 (HFF) and 4 (SSc1-3) per group; data were combined from 2 independent experiments. (*F* and *G*) *IL6* and *TNC* expression when SSc1 and HFF fibroblasts were incubated with IL6 and/or sIL6Ra for 4 h, n = 4 per group; data are representative from 2 independent experiments. (*H*) *IL6* and ECM gene expression by HFF and SSc fibroblasts in response to hyper-IL6 for 4 h, n = 4 per group. (*I*) Model of IL-6 trans-signaling in scleroderma skin grafts. tmIL6Ra is cleaved by ADAM17 (39), thereby generating the soluble receptor. IL6 expression is induced by type I interferon, which can then bind sIL6Ra and gp130 on fibroblasts to drive ECM expression along with IL6 positive feedback loop. Data are mean ± SD (*P < 0.05, **P < 0.01, ***P < 0.001, and ****P < 0.0001) analyzed with one-way ANOVA with Tukey multiple-comparisons test (*D*, *F*, and *G*) and unpaired two-tailed Student *t* test (*B*, *E*, and *H*).

To evaluate how IL-6 signaling may drive fibrosis in scleroderma skin grafts, we investigated the effects of T cell activation on expression of MHC class II and IL6 receptor alpha subunit (IL6Ra). We focused on CD4^+^ T cells because they were previously shown to shed soluble IL6Ra (sIL6Ra) (37). We activated peripheral blood CD4^+^ T cells with CD3/CD28 microbeads for three days and measured surface expression of MHC class II and membrane-bound IL6Ra (mIL6Ra) by FACS. We found that activated CD4^+^ T cells up-regulated both HLA-DR and IL6Ra on their cell surface compared to unstimulated CD4^+^ T cells (Fig. 5 *A* and *B*). sIL6Ra was detectable in the T cell supernatant of activated CD4^+^ T cells, but not unstimulated controls, demonstrating that IL6Ra is cleaved from CD4^+^ T cell surface (Fig. 5*C*). Shedding of IL6Ra from the cell surface is a characteristic feature of IL-6 trans-signaling, in which sIL6Ra binds the IL-6 cytokine and then complexes with gp130 on target cells to induce an inflammatory response (38). Our results confirm the association of T cell activation with upregulation of MHC class II and suggest CD4^+^ T cells as a source of sIL6Ra to activate IL-6 trans-signaling.

We considered whether IL-6 trans-signaling promotes fibrosis more than IL-6 cytokine alone using cultured fibroblasts. We first investigated which IL-6 signaling components are expressed in different primary fibroblast sources. We measured *IL6*, *IL6R* (soluble isoform *sIL6R* and transmembrane isoforms *tmIL6R*), and *IL6ST* (gp130) expression in human foreskin fibroblasts (HFF), adult normal dermal fibroblasts (NDF), and SSc fibroblasts. SSc fibroblasts were isolated from three patients with limited cutaneous SSc of 13 (SSc1), 4 (SSc2), and 10 (SSc3) years disease duration. SSc1 and SSc2 were not treated with immunomodulating therapy, whereas SSc3 was taking mycophenolic acid for SSc interstitial lung disease at the time of skin biopsy. Among fibroblasts, we found that HFF expressed the highest amount of IL6 and IL6ST compared to NDF and SSc fibroblasts (Fig. 5*D*). SSc1 fibroblasts expressed higher levels of sIL6R and tmIL6R than those from SSc2 and SSc3, although not as much sIL6R as HFF. Because HFF recapitulated important features of EGFR signaling in SSc in our previous studies (40) and overall showed the highest expression of IL-6-related genes, we used these and SSc fibroblasts in subsequent studies to measure their responses to recombinant type I interferon (IFNa2). We found that type I interferon induced *IL6* expression by HFF, SSc1, and SSc3 fibroblasts (Fig. 5*E*). SSc3 fibroblasts also showed higher expression of fibrotic ECM genes *TNC* and *COL1A1* in response to IFNa2, which may reflect their higher fold change in *IL6* expression compared to HFF and other SSc fibroblasts. These results align with our scRNA-Seq data showing elevated ISG signature and *IL6* expression by fibroblasts in scleroderma skin grafts. We then checked whether recombinant IL-6 or IL-6 in combination with sIL6R could induce fibrotic gene expression. We found that in both HFF and SSc1 fibroblasts, the combination of IL-6 and sIL6Ra was superior at inducing expression of *IL6* itself and the profibrotic ECM gene *TNC* compared to IL-6 alone (Fig. 5 *F* and *G*). To further assess IL-6 trans-signaling on HFF and SSc fibroblasts, we used an IL-6-IL6Ra fusion protein termed hyper-IL6 (41). We found that hyper-IL6 significantly induced expression of IL6 and TNC in HFF and SSc fibroblasts (Fig. 5*H*). Hyper-IL6 also induced type I collagen expression in HFF, but not SSc fibroblasts. Altogether, our results indicate that IL-6 trans-signaling can drive expression of multiple ECM genes, which may be regulated by sIL6Ra derived from activated CD4^+^ T cells (Fig. 5*I*). In summary, scleroderma skin grafts to humanized mice were characterized by markers of T cell activation through increased expression of MHC class II genes. Activated CD4^+^ T cells produce sIL6Ra, and in turn, sIL6Ra can bind IL-6 to drive excess ECM gene expression by fibroblasts. Thus, IL-6 trans-signaling appears to be a fundamental component driving fibrosis in our humanized mouse model of scleroderma.

## Discussion

Humanized mouse models provide an in vivo system to study mechanisms of complex human diseases. They have been used to model human tropic infections such as hepatitis C (42) and SARS-CoV-2 (43) as well as understand effects of human immune cells on tumors with patient-derived xenografts (e.g., refs. (44, 45)). Humanized mice also have high potential to better model human autoimmune diseases. Autoimmune diseases such as scleroderma and lupus occur in patients with predisposing genetic risk and a triggering event such as infection or cancer that induces immune dysregulation and disease development (1). To fully encompass these complex traits in mice, human immune cells and tissue are required. We therefore tested whether the MISTRG6 strain of humanized mice could be engrafted with healthy human skin and characterized the immune–mesenchymal interactions when transplanted with scleroderma skin. We found that in MISTRG6, but not MISTRG mice, human skin retained resident human T cells. This difference is likely due to the function of IL-6 to promote T cell differentiation and improved engraftment in MISTRG6 compared to MISTRG mice (19). Unexpectedly, however, although healthy human skin retained fibroblasts, fibroblasts were depleted from scleroderma skin and were replaced by mouse fibroblasts. Thus, MISTRG6 mice showed differential acceptance of healthy vs. fibrotic human skin xenografts. Healthy human skin grafts retain both human immune and mesenchymal cells long term, but fibrotic scleroderma skin is depleted of human mesenchymal cells.

Generation of humanized mice with skin grafts involves a coordinated sequence of events. After perinatal engraftment of mice with human HSC, fresh skin is required 8 to 10 wk later. For healthy skin grafts, we depended on good-quality human skin from discarded mastectomy and abdominoplasty surgical specimens. Unpredictable availability of fresh healthy human skin could limit the feasibility of large numbers of experiments. After obtaining human skin, an STSG was cut to reduce the thickness of human skin so that it could approximate that of the mouse recipient and remain viable. Human skin has four fibroblast populations that have heterogeneous spatial distribution (46, 47). Consequently, STSG of human skin was likely enriched in papillary dermal fibroblasts which express CD26 and WNT pathway genes (47), which are key signaling molecules in fibrosis (25, 48). The papillary dermis also contains more T cells than the reticular dermis (47), which may have facilitated their retention in the skin grafts. In sum, transplanting skin to humanized mice requires specific timing of procedures, and STSG may be enriched in T cells and fibroblast populations important in fibrosis.

A concern in humanized mice containing HSC and skin from different donors is development of graft-vs.-host disease (GvHD). GvHD occurs when there is HLA or minor histocompatibility antigen mismatch between immune cells and tissue. GvHD is characterized by elevated proinflammatory cytokines including IL-4, IL-6, IL-17, IL2Ra, and TNF (49), among which IL-6 has been proposed as key driver of disease (50, 51). To avoid bone marrow biopsy or peripheral mobilization of HSC, which can promote scleroderma (52), we engrafted MISTRG6 mice with unmatched HSC prior to transplantation of scleroderma skin grafts. Although healthy human skin was transplanted with long-term viability in MISTRG6 mice, we observed rejection of fibroblasts from scleroderma skin grafts. Scleroderma fibroblast rejection occurred independently of HSC engraftment, suggesting that it was mediated by mouse rather than human immune cells. Furthermore, engraftment with unmatched HSC reduced human IL-6 expression, which we would expect to have increased in human immune cell–mediated GvHD. Thus, although mismatched HSCs were used, our findings indicate that rejection of scleroderma fibroblasts occurred by mouse immune cells rather than human ones and suggest a loss of tolerance to a specific cell type independent of HLA.

Innate immune checkpoints may influence the capacity of humanized mice to tolerate fibroblasts in scleroderma skin transplants. The interaction between SIRPA and CD47 acts as a negative regulatory signal to inhibit macrophage-mediated phagocytosis (53). Survival of fibroblasts in fibrotic tissue requires upregulation of immunoregulatory molecules to avoid killing by immune cells. As such, inhibition of the “don’t eat me” molecule CD47 has improved skin fibrosis in mice by inducing macrophage-mediated killing of myofibroblasts (54). By extension, our results showing depletion of scleroderma fibroblasts from skin xenografts may be due to insufficient activation of mouse macrophage Sirpa by human CD47 on scleroderma fibroblasts. Although the underlying reason for loss of tolerance remains unclear, contributing factors may include somatic mutations due to phototherapy, previous immunomodulating therapies, higher oxygen demand by scleroderma skin, or higher levels of fibroblast apoptosis or senescence. We hypothesize that while human fibroblasts were tolerated in healthy skin grafts, scleroderma fibroblasts were seen as foreign and killed by mouse macrophages. Loss of scleroderma fibroblasts thus opened a cellular niche in the skin for immigration of mouse fibroblasts into scleroderma skin grafts.

IL-6 is a well-established profibrotic cytokine whose elevated expression is associated with severity of scleroderma skin fibrosis (55, 56). In MISTRG6 humanized mice, improvement in fibrosis of scleroderma skin grafts correlated with reduced expression of human IL-6. Regulation of IL-6 expression is highly complex (38), so multiple mechanisms may explain how healthy bone marrow–derived immune cells could down-regulate IL-6 expression in scleroderma skin grafts. For example, transcription of IL-6 is negatively regulated by multiple receptors including PPARa as well as estrogen and glucocorticoid receptors (57–59). Degradation of IL-6 mRNA is enhanced by RNA-binding proteins and microRNAs (60–62). Moreover, the presence of healthy human immune cells in scleroderma skin grafts may act as a sink for IL-6 protein and disrupt a positive feedback loop, such as been described for IL-6 and IL-17A (63). More studies are required to fully understand regulation of IL-6 expression in scleroderma skin grafts.

The capacity of IL-6 trans-signaling to drive fibrosis has been analyzed in mouse models of skin and lung fibrosis. In the skin, IL6R neutralization with a monoclonal antibody prevented but did not improve existing skin fibrosis in sclerodermoid GvHD model (64). In bleomycin-induced lung fibrosis via intraperitoneal injections, lung macrophages were shown to shed elevated amounts of IL6Ra, and its inhibition with recombinant gp130Fc reduced markers of myofibroblasts and improved lung function in mice (65). In vitro studies further suggested that collagen expression induced by IL-6 trans-signaling may occur indirectly through TGFβ pathway activation (66). Our scleroderma skin grafts to humanized mice support IL-6 trans-signaling as a key driver of skin fibrosis in pansclerotic morphea and align our results with recent identification of STAT4 mutations driving IL-6 expression in the childhood form of pansclerotic morphea (4). The importance of IL-6 trans-signaling was further supported by our in vitro studies of SSc fibroblasts derived from patients with limited cutaneous SSc, suggesting IL-6 as a commonly dysregulated signaling pathway in multiple scleroderma subsets. The ability of T cells to produce sIL6Ra suggests that both macrophages and CD4 T cells may be relevant cell types in promoting fibrosis in patients with scleroderma. IL-6 trans-signaling induced expression of the profibrotic gene *TNC* in addition to its previously recognized ability to induce type I collagen, thereby expanding the number of important ECM genes it regulates. In sum, through the retention of human T cells in scleroderma skin, scleroderma skin grafts to humanized mice permitted identification of IL-6 trans-signaling as a T cell–regulated process in scleroderma pathogenesis.

A limitation of our study was the use of a single donor of scleroderma skin. However, from this single donor, we were able to generate 18 skin grafts distributed onto 9 mice because of the patient’s willingness to provide an STSG that could be divided into smaller pieces. A second limitation was the unexpected depletion of human fibroblasts from scleroderma skin xenografts. As a result, we were not able to analyze immune–mesenchymal signaling between human cell types. We were interested in the effects of human T cell–derived interferon-γ on fibrosis but could not assess its role because activation of interferon-γ receptor by interferon-γ is species-specific (67). Instead, infiltrating mouse cells into scleroderma skin grafts showed a clear fibrotic signature that was modulated by HSC engraftment. This allowed us to assess whether scleroderma skin and bone marrow–derived human T cells altered fibrosis signatures via mouse cell types. Future studies and genetic alterations will be needed to achieve maintenance of human fibroblasts in scleroderma skin in MISTRG6 humanized mice.

Myeloablation followed by autologous stem cell transplantation improves event-free and overall survival of patients with severe scleroderma (68) and is associated with normalized T cell receptor diversity (69) and restoration of regulatory T cells (70). Our observations with humanized mice align with these clinical findings in that healthy bone marrow–derived hematopoietic cells improved fibrosis signatures in scleroderma skin. The main effect of healthy human bone marrow was by down-regulating IL-6 expression and its multiple downstream effects on fibrosis. We also considered whether bone marrow–derived monocytes might be antifibrotic, but their gene expression in scleroderma skin grafts did not show regulatory or antifibrotic signals. In summary, we show that healthy and scleroderma skin can be transplanted to MISTRG6 humanized mice, which maintain human T cells long term. In scleroderma skin, expression of human IL-6 corresponds with multiple pathologic features including T cell activation and ECM expression. Future work will be needed to clarify the mechanisms of human fibroblast depletion in this model.

## Materials and Methods

### Study Patients.

Skin from deidentified surgical specimens was used in experiments of healthy skin transplantation onto humanized mice. Prior to transplantation, low-quality specimens, such as having extensive stretch markings, were excluded. For STSG of scleroderma skin, a patient with pansclerotic morphea was enrolled in the approved study (Yale Human Investigation Committee #1511016816). Patient skin biopsies for SSc fibroblast isolation were part of a second approved study (Yale Human Investigation Committee #2000026608).

### Animals.

MISTRG (14) and MISTRG6 (19) mice were generated on Rag2^−/−^ IL2rg^−/−^ 129xBalb/c background with genes for human CSF1 (M-CSF), IL3, SIRPA, THPO, CSF2 (GM-CSF), and IL6 knocked into their respective mouse loci. Mice were maintained under specific pathogen-free conditions in the Yale animal facilities. Mouse experiments were conducted under a protocol approved by the Yale University Institutional Animal Care and Use Committee and in accordance with AAALAC guidelines.

### Transplantation of Human CD34+ Hematopoietic Progenitor Cells into Mice.

Recipient mice were engrafted with human hematopoietic progenitor cells as previously described (14, 71). Briefly, human fetal liver samples were cut into small fragments and treated with collagenase D (Roche) 100 ng/mL for 45 min at 37 °C. Human CD34^+^ cells from the resulting cell suspension were purified from the fetal liver by density gradient centrifugation (Lymphocyte Separation Medium, MP Biomedicals) followed by positive immunomagnetic selection with the EasySepTM Human CD34 Positive Selection Kit (StemCell). Cells were frozen in FBS (fetal bovine serum) containing 10% dimethyl sulfoxide (DMSO) and stored in liquid nitrogen. For intrahepatic engraftment, newborn 1 to 3-d-old pups were irradiated with 80 rad using a cabinet irradiator (X-RAD 320) and then injected with 10,000 fetal liver CD34^+^ cells in PBS into the liver with a 22-gauge needle (Hamilton Company).

### Skin Transplantation.

For transplantation of scleroderma skin, a 6 × 10 cm area of skin was anesthetized on the left back with 1% lidocaine with epinephrine. The site was cleansed with 3 cycles of chlorhexidine application. Using a sterilized Zimmer electric dermatome, a 5 × 7 cm by 0.75 mm thick partial-thickness skin graft was obtained and placed in cold RPMI (Roswell Park Memorial Institute) media. The wound was bandaged with Promogran Prisma (44% oxidized regenerated cellulose (ORC), 55% Collagen, and 1% silver-ORC) under Tielle nonadhesive hydropolymer and 2 large Tegaderm bandages. The patient was discharged home in good condition and tolerated the procedure without complication. The donor site was assessed weekly for 4 wk for proper healing.

### Cultured Human CD4+ T Cells and Fibroblasts.

Peripheral blood mononuclear cells (PBMCs) from deidentified healthy donors were isolated by density gradient centrifugation with Ficoll-Paque PLUS. CD4^+^ T cells were isolated from PBMCs by negative selection using the MojoSort Human CD4 T Cell Isolation Kit (BioLegend). CD4^+^ T cells were activated by incubation in Gibco RPMI 1640 Medium containing 10% FBS and 100 U/mL penicillin–streptomycin at 37 °C in 5% CO2 humidified incubator with Human T-Activator CD3/CD28 Dynabeads (Gibco) for 3 d compared to unstimulated controls in media alone. The supernatant of activated and unstimulated CD4 T cells was stored at −80 °C, and cells were immediately processed for staining. After blocking Fc receptors with TruStain FcX (BioLegend), cells were stained for HLA-DR and IL6Ra, fixed in 1% paraformaldehyde, and analyzed on the BD LSR II flow cytometer. sIL6Ra from CD4^+^ T cell supernatant was quantified using the Human IL-6R alpha Quantikine ELISA Kit (R&D Systems).

HFFs and normal adult dermal fibroblasts were purchased from ATCC (SCRC-1041 and PCS-201-010). Isolation of SSc skin fibroblasts was modified from ref. (72). Briefly, 4-mm punch biopsies were obtained from clinically involved skin on the left forearm. From each biopsy, subcutaneous fat was trimmed, and the remaining skin was minced into approximately 10 evenly sized pieces and plated on a 6-well tissue culture dish coated with 0.1% gelatin at 2 to 3 skin pieces per well. The SSc skin pieces were cultured in Dulbecco’s Modified Eagle Medium (DMEM) F12 containing 10% FBS and 100 U/mL penicillin–streptomycin 0.8 mL per well with 0.2 mL media added every 2 d. After 7 d of culture, media were increased to 2 mL total and replaced every 2 d until SSc fibroblasts were confluent for passaging.

For gene expression studies, fibroblasts were seeded in DMEM containing 1% FBS overnight at 37 °C in 5% CO2 humidified incubator. The following day, fresh media containing the indicated cytokines were added, and RNA was isolated after 4 h. Human cytokines and their concentrations included IFNa2 1,000 U/mL (BioLegend 592704), IL-6 100 ng/mL (R&D Systems 206-IL), IL6Ra 100 ng/mL (R&D Systems 227-SR), and Human IL-6/IL-6R alpha Protein Chimera 100 ng/mL (R&D Systems 8954-SR). Relative gene expression was normalized to the housekeeping gene UBC (73) and calculated using the 2^−ΔΔCt^ method (74). qPCR primers are listed below.

**Table:** 

Gene	Forward primer	Reverse primer
IL6	GCAGAAAAAGGCAAAGAATC	CTACATTTGCCGAAGAGC
IL6R	CTGGAAACTATTCATGCTACC	GACTGTTCTGAAACTTCCTC
IL6ST	AAATTGAAGCCATAGTCGTG	TTAAAATTGTGCCTTGGAGG
IL6R (75)	CATTGCCATTGTTCTGAGGTTC	GTGCCACCCAGCCAGCTATC
sIL6R (75)	GCGACAAGCCTCCCAGGTTC	GTGCCACCCAGCCAGCTATC
UBC	CGTCACTTGACAATGCAG	TGTTTTCCAGCAAAGATCAG
COL1A1	GCTATGATGAGAAATCAACCG	TCATCTCCATTCTTTCCAGG
TNC	GTGGGATCCTCTAGACATTG	GTGATCTCTCCCTCATCTTC

### Histology and Immunofluorescence of Skin Sections.

For histology, a strip of skin graft was placed in 10% neutral buffered formalin for 24 h prior to embedding in paraffin. Samples were processed at the Yale Pathology Tissue Services. Photos were taken using the Keyence BZ-X800 microscope at 10× magnification and stitched together using their software. For immunohistochemistry analysis, a strip of skin was placed in OCT, frozen, and stored at −80 °C. Using Leica CM1850 cryostat, 8 to 10-μm sections were cut, and slides were stained for the indicated human and mouse antibodies. All antibodies were purchased from BioLegend other than human CD11c (Cell Marque), human PDGFRA (Cell Signaling Technology #5241, 1/400 dilution), and mouse Pdgfra (R&D Systems #AF1062, 13 mg/mL) and used at 1/100 dilution.

**Table:** 

Human antibodies	Clone	Mouse antibodies	Clone
CD31	WM59	CD31	MEC13.3
CD146	SHM-57	CD34	HM34
CD34	561	CD45	30-F11
CD45	HI30	CD11c	N418
CD11c	5D11	CD26	H194-112
CD26	BA5b		
HLA-DR	L243		
IL6Ra	UV4		

### Single-Cell Analysis.

Each cDNA library generated from a skin graft sample was sequenced paired-end on 1 lane with 75 base-pair read length using the Illumina HiSeq 2500 System generating at least 75,000 reads per cell. The 10× genomics Cell Ranger pipeline was used to align the reads, perform clustering and gene expression analysis, and aggregate the samples with normalized read counts. All 10× experiments were completed with the same 3′ chemistry and high-throughput sequencer to avoid batch effects. CellRanger was run 3 times using 3 different genomes: mm10, hg19, and a combined mm10 and GRCh38 genome. The analysis for the mm10, hg19, and combined mm10-GRCh38 genome was conducted separately but followed the same procedure. The raw matrices from Cell Ranger were processed and analyzed using the Seurat version 3 R toolkit for single-cell genomics (76, 77). For the matrices aligned to the mm10 genome and the combined mm10-GRCh38 genome, low-quality cells with a high mitochondrial percentage of over 7.5% were filtered out. For the matrices aligned to the hg19 genome, low-quality cells with a mitochondrial percentage of over 10% were filtered out. The data were then normalized (log normalized using the default scaling factor of 10,000) and scaled. Principal components were calculated and the data were visualized by UMAP embedding. After determining the clusters, canonical marker genes were used to identify the cell types present. Once the cell types were identified, we used DAseq (26) to identify the regions that were DA between HSC engraftment and unengrafted samples. From DAseq, identified cell types with DA regions and then analyzed differential gene expression in those cell types using Seurat. Pathway analysis of differentially expressed genes with adjusted *P* value of less than 0.05 was completed with Metascape (78).

### Statistical Analysis.

Differential gene expression analysis of scRNA-Seq data was performed using Cell Ranger and Loupe Cell Browser software (10× Genomics), which uses a variant of the negative binomial exact test from sSeq and the asymptotic beta test in edgeR depending on sample size (79, 80). Statistical analysis and graphs were generated using GraphPad Prism v9. Pairwise comparisons were analyzed using the two-tailed Student *t* test and multiple comparison with one-way ANOVA. For all graphs, *P < 0.05, **P < 0.01, ***P < 0.001, and ****P < 0.0001.

## Data Availability

Raw scRNA sequencing data generated in this study were deposited to the NCBI Gene Expression Omnibus under the accession number GSE240009 (81).
